# *N*-Phenyl-*tert*-butane­sulfinamide

**DOI:** 10.1107/S1600536809028633

**Published:** 2009-07-29

**Authors:** Mritunjoy Datta, Alan J. Buglass, Mark R. J. Elsegood

**Affiliations:** a Department of Chemistry, Korea Advanced Institute of Science and Technology, Daejeon 305-701, Republic of Korea; b Chemistry Department, Loughborough University, Loughborough LE11 3TU, England

## Abstract

In the racemic title compound, C_10_H_15_NOS, the packing exhibits centrosymmetric pairs of mol­ecules linked by N—H⋯O=S hydrogen bonds in a head-to-tail fashion. The N—C_ar­yl_ bond [1.4083 (12) Å] is considerably shorter than the N—C_alk­yl_ bonds typically found in *N*-alkyl­alkanesulfinamides (1.470–1.530 Å).

## Related literature

For *N*-aryl­alkanesulfinamides, see: Datta *et al.* (2008 ▶) and for cyclic *N*-aryl­alkanesulfinamides (sultims), see: Schulze *et al.* (2005 ▶). For *N*-alkyl­alkanesulfinamides, see: Sato *et al.* (1975 ▶); Schuckmann *et al.* (1978 ▶); Ferreira *et al.* (2005 ▶). For the synthesis, see: Stretter *et al.* (1969 ▶).
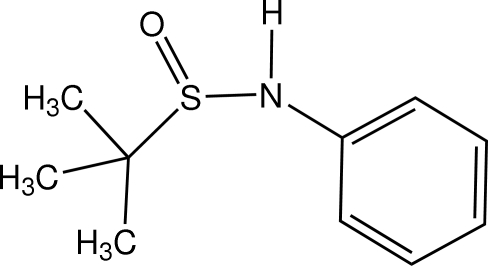


## Experimental

### 

#### Crystal data

C_10_H_15_NOS
*M* *_r_* = 197.29
Monoclinic, 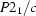

*a* = 7.4822 (3) Å
*b* = 15.7881 (6) Å
*c* = 8.8333 (4) Å
β = 99.3865 (6)°
*V* = 1029.50 (7) Å^3^
*Z* = 4
Mo *K*α radiation
μ = 0.28 mm^−1^
*T* = 150 K
0.54 × 0.49 × 0.39 mm

#### Data collection

Bruker APEXII CCD diffractometer
Absorption correction: multi-scan (*SADABS*; Sheldrick, 2007 ▶) *T*_min_ = 0.866, *T*_max_ = 0.900
12022 measured reflections
3150 independent reflections
2861 reflections with *I* > 2σ(*I*)
*R*_int_ = 0.020

#### Refinement

*R*[*F* ^2^ > 2σ(*F* ^2^)] = 0.034
*wR*(*F* ^2^) = 0.092
*S* = 1.05
3150 reflections
125 parameters
1 restraint
H atoms treated by a mixture of independent and constrained refinement
Δρ_max_ = 0.38 e Å^−3^
Δρ_min_ = −0.36 e Å^−3^

### 

Data collection: *APEX2* (Bruker, 2006 ▶); cell refinement: *SAINT* (Bruker, 2006 ▶); data reduction: *SAINT*; program(s) used to solve structure: *SHELXS97* (Sheldrick, 2008 ▶); program(s) used to refine structure: *SHELXL97* (Sheldrick, 2008 ▶); molecular graphics: *SHELXTL* (Sheldrick, 2008 ▶); software used to prepare material for publication: *SHELXTL* and local programs.

## Supplementary Material

Crystal structure: contains datablocks I, global. DOI: 10.1107/S1600536809028633/zs2002sup1.cif

Structure factors: contains datablocks I. DOI: 10.1107/S1600536809028633/zs2002Isup2.hkl

Additional supplementary materials:  crystallographic information; 3D view; checkCIF report

## Figures and Tables

**Table 1:** Hydrogen-bond geometry (Å, °)

*D*—H⋯*A*	*D*—H	H⋯*A*	*D*⋯*A*	*D*—H⋯*A*
N1—H1⋯O1^i^	0.808 (12)	2.085 (12)	2.8882 (11)	173.1 (14)

Symmetry code: (i) 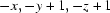.

**Fig. 1.**
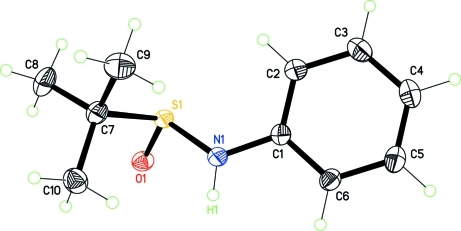
Molecular structure of (I), with atom labels and 50% probablility displacement ellipsoids for non-H atoms.

**Fig. 2.**
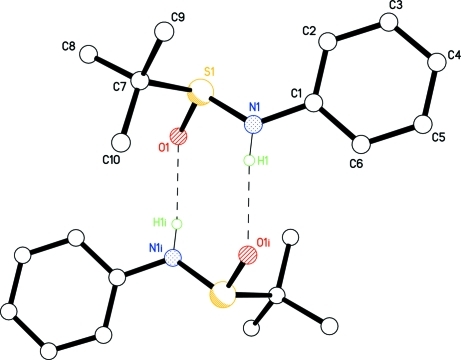
Intermolecular hydrogen bonding between a pair of opposite enantiomers of (I) in the crystal packing. Symmetry code i = -x, -y + 1, -z + 1.

## supplementary crystallographic information

### Comment

The molecular structure of (I) (Fig. 1) exhibits a short *N*—(aryl)C Bond (1.4083 (12) Å), like that in *N*-phenyladamantane-1-sulfinamide (1.409 (2) Å) (Datta *et al.*, 2008), and in contrast with *N*—(alkyl)C bonds in *N*-alkylalkanesulfinamides (1.470–1.530 Å) (Sato *et al.*, 1975; Schuckmann *et al.*, 1978; Ferreira *et al.*, 2005). Otherwise, molecular geometry is similar to other sulfinamides. The molecules of (I) in the crystal lattice are linked by pairs of N—H···O=S hydrogen bonds (Fig. 2 and Table 1). There is no evidence of weak intermolecular C—H···O=S hydrogen bonds, as in the packing of *N*-phenyladamantane-1-sulfinamide (Datta *et al.*, 2008). The crystal system and space group for (I) and *N*-phenyladamantane-1-sulfinamide are the same (namely monoclinic and *P*2_1_/*c*, respectively).

### Experimental

Compound (I) was prepared by the method of Stretter *et al.* (1969), using *tert*-butanesulfinyl chloride (702.5 mg, 5 mmol) and aniline (930 mg, 10 mmol) in dry chloroform (30 ml). After 6 h (with TLC monitoring) the white solid amine salt was filtered off and the solvent was removed under reduced pressure. Column chromatography (silica gel, dichloromethane) provided (I) as white crystals (950 mg, 96%), m.p. 376–377 K. Single crystals suitable for X-ray analysis were obtained by evaporation of a solution of (I) in dichloromethane at room temperature. Spectroscopic analysis: FTIR (KBr) (cm^-1^) 3015, 2599, 2330, 1496, 1469, 1420, 1370, 1274, 1068,1026, 888, 859. ^1^H NMR (400 MHz, CDCl_3_ p.p.m. with respect to TMS) δ 7.26–7.22 (m, 2H), 7.00–6.98 (m, 3H), 5.48 (bs, 1H), 1.30 (s, 9H). ^13^C NMR (100 MHz, CDCl_3_ p.p.m. with respect to TMS) δ 142.0, 129.4, 123.0, 118.4, 56.5, 22.4. EIMS m/*z* (%) 197 (*M*^+^, 18) 141 (100), 140 (*M*^+^-*^t^*Bu, 28), 105 (*M*^+^—PhNH, 86), 92 (*M*^+^-*^t^*BuSO, 72), 78 (77), 57 (93).

### Refinement

H atoms were located in a difference Fourier map and refined geometrically using a riding model except for N*H*for which the coordinates were freely refined. Bond lengths and displacement parameters were constrained as follows: C—H = 0.95–0.98 Å and *U_iso_*(H) = 1.2 (1.5 for CH_3_) times *U_eq_*(C, N).

### Crystal data

**Table:**

C_10_H_15_NOS	*F*(000) = 424
*M**_r_* = 197.29	*D*_x_ = 1.273 Mg m^−^^3^
Monoclinic, *P*2_1_/*c*	Melting point = 376–377 K
Hall symbol: -P 2ybc	Mo *K*α radiation, λ = 0.71073 Å
*a* = 7.4822 (3) Å	Cell parameters from 6597 reflections
*b* = 15.7881 (6) Å	θ = 2.3–30.5°
*c* = 8.8333 (4) Å	µ = 0.28 mm^−^^1^
β = 99.3865 (6)°	*T* = 150 K
*V* = 1029.50 (7) Å^3^	Block, colourless
*Z* = 4	0.54 × 0.49 × 0.39 mm

### Data collection

**Table:**

Bruker APEXII CCD diffractometer	3150 independent reflections
Radiation source: fine-focus sealed tube	2861 reflections with *I* > 2σ(*I*)
graphite	*R*_int_ = 0.020
ω rotation with narrow frames scans	θ_max_ = 30.6°, θ_min_ = 2.7°
Absorption correction: multi-scan (*SADABS*; Sheldrick, 2007)	*h* = −10→10
*T*_min_ = 0.866, *T*_max_ = 0.900	*k* = −22→22
12022 measured reflections	*l* = −12→12

### Refinement

**Table:**

Refinement on *F*^2^	Secondary atom site location: difference Fourier map
Least-squares matrix: full	Hydrogen site location: inferred from neighbouring sites
*R*[*F*^2^ > 2σ(*F*^2^)] = 0.034	H atoms treated by a mixture of independent and constrained refinement
*wR*(*F*^2^) = 0.092	*w* = 1/[σ^2^(*F*_o_^2^) + (0.0516*P*)^2^ + 0.2529*P*] where *P* = (*F*_o_^2^ + 2*F*_c_^2^)/3
*S* = 1.05	(Δ/σ)_max_ = 0.002
3150 reflections	Δρ_max_ = 0.38 e Å^−^^3^
125 parameters	Δρ_min_ = −0.36 e Å^−^^3^
1 restraint	Extinction correction: *SHELXL97* (Sheldrick, 2008), Fc^*^=kFc[1+0.001xFc^2^λ^3^/sin(2θ)]^-1/4^
Primary atom site location: structure-invariant direct methods	Extinction coefficient: 0.020 (3)

### Special details

**Table:**

Geometry. All e.s.d.'s (except the e.s.d. in the dihedral angle between two l.s. planes) are estimated using the full covariance matrix. The cell e.s.d.'s are taken into account individually in the estimation of e.s.d.'s in distances, angles and torsion angles; correlations between e.s.d.'s in cell parameters are only used when they are defined by crystal symmetry. An approximate (isotropic) treatment of cell e.s.d.'s is used for estimating e.s.d.'s involving l.s. planes.
Refinement. Refinement of *F*^2^ against ALL reflections. The weighted *R*-factor *wR* and goodness of fit *S* are based on *F*^2^, conventional *R*-factors *R* are based on *F*, with *F* set to zero for negative *F*^2^. The threshold expression of *F*^2^ > σ(*F*^2^) is used only for calculating *R*-factors(gt) *etc*. and is not relevant to the choice of reflections for refinement. *R*-factors based on *F*^2^ are statistically about twice as large as those based on *F*, and *R*- factors based on ALL data will be even larger.

### Fractional atomic coordinates and isotropic or equivalent isotropic displacement parameters (Å^2^)

**Table:**

	*x*	*y*	*z*	*U*_iso_*/*U*_eq_	
O1	−0.06049 (10)	0.60738 (4)	0.41648 (9)	0.02295 (16)	
S1	0.10835 (3)	0.601595 (13)	0.34614 (3)	0.01896 (9)	
N1	0.17155 (13)	0.50050 (5)	0.35165 (10)	0.02446 (18)	
H1	0.1463 (19)	0.4727 (9)	0.4219 (15)	0.029*	
C1	0.19492 (12)	0.45439 (6)	0.21979 (11)	0.01998 (18)	
C2	0.24483 (16)	0.49296 (7)	0.09080 (12)	0.0281 (2)	
H2	0.2589	0.5527	0.0877	0.034*	
C3	0.27381 (17)	0.44344 (7)	−0.03327 (13)	0.0318 (2)	
H3	0.3053	0.4700	−0.1218	0.038*	
C4	0.25757 (15)	0.35617 (7)	−0.02991 (13)	0.0295 (2)	
H4	0.2790	0.3230	−0.1148	0.035*	
C5	0.20955 (14)	0.31769 (6)	0.09922 (12)	0.0257 (2)	
H5	0.1990	0.2578	0.1029	0.031*	
C6	0.17690 (13)	0.36629 (6)	0.22303 (11)	0.02134 (18)	
H6	0.1422	0.3396	0.3101	0.026*	
C7	0.29072 (13)	0.64579 (6)	0.48984 (11)	0.02172 (18)	
C8	0.24368 (16)	0.73950 (7)	0.50283 (14)	0.0316 (2)	
H8A	0.3444	0.7687	0.5668	0.047*	
H8B	0.1342	0.7448	0.5497	0.047*	
H8C	0.2223	0.7650	0.4003	0.047*	
C9	0.46486 (15)	0.63537 (8)	0.42278 (14)	0.0312 (2)	
H9A	0.5658	0.6613	0.4919	0.047*	
H9B	0.4512	0.6632	0.3224	0.047*	
H9C	0.4894	0.5750	0.4110	0.047*	
C10	0.30068 (15)	0.60073 (6)	0.64357 (12)	0.0265 (2)	
H10A	0.3290	0.5408	0.6314	0.040*	
H10B	0.1839	0.6057	0.6794	0.040*	
H10C	0.3956	0.6267	0.7188	0.040*	

### Atomic displacement parameters (Å^2^)

**Table:**

	*U*^11^	*U*^22^	*U*^33^	*U*^12^	*U*^13^	*U*^23^
O1	0.0218 (3)	0.0215 (3)	0.0264 (4)	0.0011 (2)	0.0067 (3)	0.0021 (2)
S1	0.02263 (13)	0.01561 (12)	0.01895 (13)	0.00052 (7)	0.00429 (8)	0.00155 (7)
N1	0.0397 (5)	0.0153 (3)	0.0206 (4)	0.0013 (3)	0.0114 (3)	0.0012 (3)
C1	0.0214 (4)	0.0187 (4)	0.0202 (4)	0.0003 (3)	0.0045 (3)	−0.0011 (3)
C2	0.0397 (6)	0.0210 (4)	0.0254 (5)	−0.0014 (4)	0.0112 (4)	0.0010 (4)
C3	0.0422 (6)	0.0327 (6)	0.0224 (5)	0.0011 (4)	0.0111 (4)	0.0006 (4)
C4	0.0324 (5)	0.0316 (5)	0.0251 (5)	0.0014 (4)	0.0063 (4)	−0.0080 (4)
C5	0.0247 (5)	0.0218 (4)	0.0304 (5)	−0.0010 (3)	0.0043 (4)	−0.0060 (4)
C6	0.0207 (4)	0.0186 (4)	0.0252 (4)	−0.0012 (3)	0.0053 (3)	−0.0006 (3)
C7	0.0218 (4)	0.0186 (4)	0.0242 (4)	0.0006 (3)	0.0022 (3)	−0.0001 (3)
C8	0.0352 (5)	0.0183 (4)	0.0387 (6)	−0.0002 (4)	−0.0013 (4)	−0.0037 (4)
C9	0.0230 (5)	0.0366 (6)	0.0346 (6)	0.0004 (4)	0.0065 (4)	0.0042 (4)
C10	0.0290 (5)	0.0278 (5)	0.0220 (5)	0.0026 (4)	0.0020 (4)	0.0006 (3)

### Geometric parameters (Å, °)

**Table:**

O1—S1	1.4988 (7)	C5—H5	0.9500
S1—N1	1.6632 (9)	C6—H6	0.9500
S1—C7	1.8426 (10)	C7—C10	1.5241 (14)
N1—C1	1.4083 (12)	C7—C9	1.5255 (14)
N1—H1	0.808 (12)	C7—C8	1.5294 (14)
C1—C2	1.3954 (13)	C8—H8A	0.9800
C1—C6	1.3982 (12)	C8—H8B	0.9800
C2—C3	1.3918 (15)	C8—H8C	0.9800
C2—H2	0.9500	C9—H9A	0.9800
C3—C4	1.3838 (17)	C9—H9B	0.9800
C3—H3	0.9500	C9—H9C	0.9800
C4—C5	1.3903 (15)	C10—H10A	0.9800
C4—H4	0.9500	C10—H10B	0.9800
C5—C6	1.3898 (13)	C10—H10C	0.9800
			
O1—S1—N1	107.53 (4)	C10—C7—C9	112.02 (8)
O1—S1—C7	105.72 (4)	C10—C7—C8	111.29 (9)
N1—S1—C7	99.69 (5)	C9—C7—C8	110.80 (9)
C1—N1—S1	123.02 (7)	C10—C7—S1	111.11 (7)
C1—N1—H1	115.5 (10)	C9—C7—S1	105.97 (7)
S1—N1—H1	116.3 (10)	C8—C7—S1	105.33 (7)
C2—C1—C6	119.36 (9)	C7—C8—H8A	109.5
C2—C1—N1	122.39 (8)	C7—C8—H8B	109.5
C6—C1—N1	118.16 (8)	H8A—C8—H8B	109.5
C3—C2—C1	119.65 (10)	C7—C8—H8C	109.5
C3—C2—H2	120.2	H8A—C8—H8C	109.5
C1—C2—H2	120.2	H8B—C8—H8C	109.5
C4—C3—C2	121.12 (10)	C7—C9—H9A	109.5
C4—C3—H3	119.4	C7—C9—H9B	109.5
C2—C3—H3	119.4	H9A—C9—H9B	109.5
C3—C4—C5	119.21 (9)	C7—C9—H9C	109.5
C3—C4—H4	120.4	H9A—C9—H9C	109.5
C5—C4—H4	120.4	H9B—C9—H9C	109.5
C6—C5—C4	120.43 (9)	C7—C10—H10A	109.5
C6—C5—H5	119.8	C7—C10—H10B	109.5
C4—C5—H5	119.8	H10A—C10—H10B	109.5
C5—C6—C1	120.21 (9)	C7—C10—H10C	109.5
C5—C6—H6	119.9	H10A—C10—H10C	109.5
C1—C6—H6	119.9	H10B—C10—H10C	109.5
			
O1—S1—N1—C1	123.13 (8)	C4—C5—C6—C1	−1.02 (15)
C7—S1—N1—C1	−126.86 (8)	C2—C1—C6—C5	0.40 (14)
S1—N1—C1—C2	28.58 (14)	N1—C1—C6—C5	−176.09 (9)
S1—N1—C1—C6	−155.05 (8)	O1—S1—C7—C10	54.89 (8)
C6—C1—C2—C3	0.76 (16)	N1—S1—C7—C10	−56.55 (8)
N1—C1—C2—C3	177.09 (10)	O1—S1—C7—C9	176.79 (7)
C1—C2—C3—C4	−1.32 (18)	N1—S1—C7—C9	65.35 (7)
C2—C3—C4—C5	0.70 (17)	O1—S1—C7—C8	−65.73 (8)
C3—C4—C5—C6	0.48 (16)	N1—S1—C7—C8	−177.17 (7)

### Hydrogen-bond geometry (Å, °)

**Table:**

*D*—H···*A*	*D*—H	H···*A*	*D*···*A*	*D*—H···*A*
N1—H1···O1^i^	0.81 (1)	2.09 (1)	2.8882 (11)	173 (1)

Symmetry codes: (i) −*x*, −*y*+1, −*z*+1.
